# A three-year follow-up study evaluating clinical utility of exome sequencing and diagnostic potential of reanalysis

**DOI:** 10.1038/s41525-020-00144-x

**Published:** 2020-09-10

**Authors:** Jasmine L. F. Fung, Mullin H. C. Yu, Shushu Huang, Claudia C. Y. Chung, Marcus C. Y. Chan, Sander Pajusalu, Christopher C. Y. Mak, Vivian C. C. Hui, Mandy H. Y. Tsang, Kit San Yeung, Monkol Lek, Brian H. Y. Chung

**Affiliations:** 1 Department of Paediatrics and Adolescent Medicine, LKS Faculty of Medicine, The University of Hong Kong, Hong Kong SAR, China; 2 Department of Genetics, Yale School of Medicine, New Haven, CT 06510 USA; 3 Department of Geriatrics, The Affiliated Hospital of Nantong University, Nantong, 210029 China; 4 The First School of Clinical Medicine, Nanjing Medical University, Nanjing, 226001 China; 5 Department of Clinical Genetics, United Laboratories, Tartu University Hospital, Tartu, Estonia; 6 Department of Clinical Genetics, Institute of Clinical Medicine, University of Tartu, Tartu, Estonia

**Keywords:** Clinical genetics, Paediatrics, Genetics research, Outcomes research

## Abstract

Exome sequencing (ES) has become one of the important diagnostic tools in clinical genetics with a reported diagnostic rate of 25–58%. Many studies have illustrated the diagnostic and immediate clinical impact of ES. However, up to 75% of individuals remain undiagnosed and there is scarce evidence supporting clinical utility beyond a follow-up period of >1 year. This is a 3-year follow-up analysis to our previous publication by Mak et al. (*NPJ Genom. Med.* 3:19, 2018), to evaluate the long-term clinical utility of ES and the diagnostic potential of exome reanalysis. The diagnostic yield of the initial study was 41% (43/104). Exome reanalysis in 46 undiagnosed individuals has achieved 12 new diagnoses. The additional yield compared with the initial analysis was at least 12% (increased from 41% to at least 53%). After a median follow-up period of 3.4 years, change in clinical management was observed in 72.2% of the individuals (26/36), leading to positive change in clinical outcome in four individuals (11%). There was a minimum healthcare cost saving of HKD$152,078 (USD$19,497; €17,282) annually for these four individuals. There were a total of six pregnancies from five families within the period. Prenatal diagnosis was performed in four pregnancies; one fetus was affected and resulted in termination. None of the parents underwent preimplantation genetic diagnosis. This 3-year follow-up study demonstrated the long-term clinical utility of ES at individual, familial and health system level, and the promising diagnostic potential of subsequent reanalysis. This highlights the benefits of implementing ES and regular reanalysis in the clinical setting.

## Introduction

Since the advancement of next-generation sequencing (NGS) technology, exome sequencing (ES), and genome sequencing (GS) have received increasing attention and application in clinical diagnostics, owing to its comprehensive and timely diagnostic capacity. The diagnostic and clinical utility of ES/GS have been well-studied and proven to be greater than chromosomal microarray or other conventional molecular testing in children with suspected genetic disorders^1–5^. However, in practice, ES is more widely used than GS because ES has a lower cost, while covering all known exons with majority of the pathogenic variants. Although there are relatively few studies on the diagnostic utility of GS, a meta-analysis of these studies showed that clinically there is a minimal difference between using GS and ES methods^5^.

The mean diagnostic rate of ES is estimated to be 36%, ranging from 25 to 58%, depending on the patient selection criteria and the disease type^3,5–8^. ES can be a powerful diagnostic tool compared to conventional methods, but a large proportion of individuals remain undiagnosed. One reason for this may be owing to the clinical and genetic heterogeneity of rare diseases. On the other hand, causal variants in the exome may be unrecognized due to limitations in the analytical methods or inadequate knowledge in the literature regarding the disease genetics. These unsolved cases fall into the “experimental maze”, where the attempt to make a diagnosis becomes a trial of novel approaches. Frésard and Montgomery discussed multiple strategies to address this issue, including exome reanalysis, GS, long-read sequencing, and other omics studies^9^. Among these methods, reanalysis of the exome is the most accessible and inexpensive. Multiple studies with predominantly Caucasian individuals have shown that the diagnostic rate of reanalysis ranges from 6 to 47%^10–23^. Stark et al. has estimated that 18-months is the most cost-effective time point to perform reanalysis^19^. In comparison, other approaches such as GS as a second-tier test for negative ES are less well-studied.

There is no doubt that a positive genetic diagnosis could provide further information to aid clinical decisions and patient management. The change in medical management is thus commonly used as an indicator of clinical utility, where a measure of outcome can be captured at three instances: (i) the return of results, (ii) documented changes to clinical management, and (iii) measures of long-term clinical outcome^24^. While many studies focused on describing the changes in acute management brought about by a positive genetic diagnosis, very few have assessed the long-term impact by longitudinal follow-up of >1 year^3,19,25–29^. Furthermore, the impact on reproductive decisions for both the affected individuals and their families is often limited. Lastly, there is also scarcity of health economic evidence on long-term follow-up after ES, which is important to guide the healthcare policy development^30^.

Based on our previously published cohort of predominant Chinese pediatric patients who underwent ES in 2013–2017, the current study aims to address two main questions: (i) the potential of ES reanalysis in making further diagnoses; and (ii) the long-term outcome of initial ES-positive individuals, measured at the individual level (change in clinical management and/or outcome); the family level (effect on reproductive decision making of the parents); and health system level (healthcare utilization and associated costs).

## Results

### Improved diagnostic yield through exome reanalysis

All undiagnosed individuals in the initial analysis (*n* = 61) were invited to participate in the reanalysis. Of the 61 undiagnosed individuals, 46 of them consented for reanalysis (13 were unable to be contacted and two refused to participate). Thus, for this study, our reanalysis is limited to these 46 individuals summarized in Supplementary Table 1. ES was performed on stored DNA of 34 individuals, and reanalysis on existing raw data was performed in 12 individuals. Twenty-six were analyzed as singletons, one as duo (proband and affected sibling), and 19 as trios. The average exome read coverage was 76.2×, while the percent of targeted bases over 20× coverage was 87.1%.

An additional 12 diagnoses were made through ES reanalysis (26% of the reanalyzed cohort), boosting the overall diagnostic rate from 41% (43/104) to at least 53% (55/104). Nine are autosomal dominant conditions, with heterozygous pathogenic/likely pathogenic variants identified in *ATP1A3*, *COL11A1*, *GNB1*, *MN1*, *MFN2*, *PACS1*, *PTPN11*, and *SPTAN1* (two probands); three are autosomal recessive conditions (*COQ7*, *PRF1*, and *SKIV2L*). The summary of the findings is summarized in Table 1 and Supplementary Table 2.

**Table 1:** Genetic diagnoses made by WES reanalysis and the reason for positive result in 12 patients.

ID	Sample Type	Singleton/ Trios	Gene	Variant(s)	Segregation	Diagnosis (OMIM number)	Inheritance	Reasons for diagnosis					
								U	P	A	S	B	R
U018	DNA	Trios	*ATP1A3*	NM_152296.5:c.954 C > G, p.(Ile318Met)	De novo	Dystonia-12 (#128235); alternating hemiplegia of childhood 2 (#614820)	AD	●	○	○			
U022	DNA	Singleton	*PACS1*	NM_018026.4:c.607 C > T, p.(Arg203Trp)	De novo^a^	Schuurs-Hoeijmakers syndrome (#615009)	AD	●					
U036	DNA	Trios	*MN1*	NM_002430.3:c.3870_3879dupTGACGCCAAG, p.(Ala1294Ter)	De novo	*MN1* C-terminal truncation (MCTT) syndrome (#618774)	AD						●
U043	DNA	Trios	*PTPN11*	NM_002834.5:c.5 C > T, p.(Thr2Ile)	De novo	Noonan syndrome (#163950)	AD		○		●		
U045	DNA	Trios	*SKIV2L*	NM_006929.5:c.1404-2 A > G; NM_006929.5:c.1647 + 1 G > A	Inherited	Trichohepatoenteric syndrome 2 (#614602)	AR				●		
U057	DNA	Duos	*PRF1*	NM_005041.5:c.1018 G > A, p.(Asp340Asn) (homozygous)	Inherited	Hemophagocytic lymphohistiocytosis, familial, 2 (#603553)	AR		○	●			
U066	DNA	Singleton	*MFN2*	NM_001127660.1:c.707 C > T, p.(Thr236Met)	Unknown	Charcot-Marie-Tooth disease, axonal, type 2A2A (#609260); hereditary motor and sensory neuropathy VIA (#601152)	AD	●					
U071	DNA	Singleton	*COL11A1*	NM_001854.4:c.3115 G > A, p.(Gly1039Ser)	Unknown	Stickler syndrome, type II (#604841); Marshall syndrome (#154780)	AD		●				
U075	DNA	Singleton	*SPTAN1*	NM_001130438.3: c.(?_1225)_(1572_?)del (exon 10–12 deletion)	De novo^a^	Epileptic encephalopathy, early infantile, 5 (#613477)	AD					●	
U077	Data	Trios	*SPTAN1*	NM_001130438.3:c.4828 C > T, p.(Arg1610Trp)	De novo	Epileptic encephalopathy, early infantile, 5 (#613477)	AD	●		○			
U086	Data	Singleton	*GNB1*	NM_002074.5:c.239 T > C, p.(Ile80Thr)	Unknown	Intellectual disability, autosomal dominant 42 (#616973)	AD	●					
U094	Data	Trios	*COQ7*	NM_016138.5:c.599_600delinsTAATGCATC, p.(Lys200Ilefs*56); NM_016138.5:c.319 C > T, p.(Arg107Trp)	Inherited	Coenzyme Q_10_ deficiency, primary, 8 (#616733)	AR	●					
							Count:	6	4	3	2	1	1
							Percentage:	50%	33%	25%	17%	8%	8%

The circles in the table indicate the reasons for reanalysis positive and the filled circles (●) denote the major contributing factor.

*U* update in medical literature/ database, *P* patient has phenotype update or has atypical clinical presentation, *A* additional sequencing for trios/affected family member(s), *S* improved sequencing, *B* improved bioinformatics*, R* research collaboration (new gene/ syndrome), *AD* autosomal dominant, *AR* autosomal recessive.

^a^Targeted parental testing was performed to confirm the segregation.

Among the 12 new positive diagnoses, the majority (6/12; 50%) of individuals harbored variants in genes that had weak/no gene–disease association during the time of initial ES. The pathogenicity was established in reanalysis with increasing evidence from the latest literature.

For individual U018, a novel pathogenic variant was identified in *ATP1A3*. This gene has been known and thought to be the cause of three distinct diseases since 2004: rapid-onset dystonia Parkinsonism (RDP), alternating hemiplegia of childhood (AHC), and CAPOS (cerebellar ataxia, areflexia, pes cavus, optic atrophy, and sensorineural hearing loss) syndrome^31–34^. It was not until 2014 that it was suggested that they belong to the same spectrum of disease^35^. An updated review of the growing evidence of patients presenting with intermediate/mixed/atypical phenotypes was published in 2018^35,36^. This clinical spectrum of disease is now summarized as *ATP1A3*-related neurological disorder, and our patient’s phenotype belongs to mixed phenotype of RDP and AHC.

ES reanalysis diagnosed individual U022 with Schuss-Hoeijmakers syndrome caused by a pathogenic variant in the gene *PACS1. PACS1* was first discovered in 2012 in which two individuals who shared similar phenotypes were found to harbor the same variant^37^. The sample size remained limited until a case series of 19 individuals were reported in 2016, while the initial ES of U022 was performed in 2014^38^.

U077 presented with global developmental delay, hypotonia, and MRI finding of mega cisterna magna. Exome reanalysis revealed a de novo missense mutation in *SPTAN1* that explained his phenotype. This gene was first reported to be associated with a distinctive form of West syndrome with hypomyelination in 2008, later classified as early infantile epileptic encephalopathy^39,40^. Syrbe et al. reported six individuals with milder phenotypes of less severe intellectual disability with or without epilepsy; in particular, the variant identified in U077 was also reported^41^.

An *MFN2* variant has been identified in individual U066. This variant was detected in the initial analysis performed in 2016, but after extensive review was concluded as unsolved, as he did not have any symptoms of Charcot-Marie-Tooth (CMT) disease. Rather, he presented with intellectual disability, behavioral problems, and seizure. After independent reanalysis, the same variant has been identified and classified as likely pathogenic, and now, there is stronger variant–disease association. This variant has been reported as pathogenic/likely pathogenic in multiple individuals with CMT disease in ClinVar, in which multiple submitters providing assertion criteria provided the same interpretation (two stars evidence). Upon discussion with neurologist and further literature review, there are individuals carrying pathogenic variant in *MFN2* reported to present with developmental delay and other central nervous system involvement in addition to neuropathies^42^. Variable age of onset (up to 50 years old), expressivity and incomplete penetrance have been observed^43–45^. Therefore, although individual U066 did not present with the typical phenotype of neuropathy of CMT disease, it may due to late disease-onset (U066 is currently 14 years old), reduced expressivity or non-penetrance. This likely pathogenic variant is reported as it may have an implication to the further care and management.

During the initial ES in 2016, the association of *COQ7* and coenzyme Q_10_ (CoQ_10_) deficiency was limited as there was only one article reporting an individual with homozygous variant in 2015^46^. After reanalysis, individual U094 became the third case reported with this diagnosis, caused by compound heterozygous variants in *COQ7*^47^. He has clinical phenotype compatible to CoQ_10_ deficiency and further skin fibroblast testing showed a reduction in CoQ_10_ level and decreased combined complex II + III activity, supporting that the biallelic variants may contribute to U094’s phenotype^47^.

Lastly, *GNB1* was a new gene discovered near the time of the initial ES of individual U086. The first paper on *GNB1* was published in May 2016, while initial ES was requested in March 2016 (report issued on in June 2016)^48^. This variant has been reported as a de novo pathogenic variant in multiple patients with similar phenotype, and considered as a “mutation hotspot” within the gene^48,49^. Therefore, the genetic diagnosis is established in U086.

New diagnoses were made in four patients because of an update on phenotype that became more apparent with age or recognition of nonclassical/atypical symptoms that masked the core features when the patient was first assessed (33%).

For example, as discussed above, individual U018 was presented with mixed phenotype of RDP and AHC, which is atypical to the early reports on the association between *ATP1A3* and diseases.

Reanalysis yielded the diagnosis of Noonan syndrome in individual U043, who first presented as a new born with congenital arthrogryposis multiplex rather than typical features of Noonan syndrome. In retrospect, U043 has mild developmental delay and the facial feature resembling Noonan syndrome became more apparent upon clinical evaluation at 4 years of age. Interestingly, multiple joint contractures only occur in 4% of patients with Noonan syndrome^50^.

Individual U057 was born from a consanguineous family, presented with developmental delay and epilepsy, and later passed away due to acute deterioration after high swinging fever with multi-organ failure. Initial ES at that time was unrevealing. Reanalysis found a homozygous variant in the *PRF1* gene, which is associated with hemophagocytic lymphohistiocytosis (HLH) type 2. His predominant neurological manifestation at onset was nonclassical for HLH, but in retrospect, the clinical course is compatible and the diagnosis was supported by the additional phenotype of T-cell lymphoma and intracranial lesion from postmortem examination.

Lastly, for individual U071, the diagnosis was Stickler syndrome/Marshall syndrome (*COL11A1*). In addition to his connective tissue problem explainable by the diagnosis, he had hypotonia during infancy, gross motor delay, Marfanoid habitus, scoliosis, prominent aortic sinus, and limited intelligence that may have prompted clinicians to consider other differential diagnosis, such as Marfan syndrome, Sprinzten Golderg syndrome, or neuromuscular diseases. The diagnosis of *COL11A1* could only partially explain his phenotype of connective tissue disorder. We have evaluated other genes in relation to these additional phenotypes, but so far no other candidate genetic variant has been identified. It is uncertain if there is other genetic factor contributing to his clinical presentation.

Additional sequencing for trios (individuals U018 and U077) and other similarly affected family member (U057) aided analysis in three patients (25%). The variants identified in U018 and U077 were found de novo, which trio analyses allowed immediate delineation of the inheritance pattern and support the pathogenicity. For U057, reanalysis was initiated when a younger sibling presented with similar phenotype of development regression and status epilepticus. The reanalysis with the affected sibling found homozygous variants in *PRF1* in both siblings.

Improved sequencing technology also contributed to the increased yield (17%), where two variants had increased sequence coverage with re-sequencing: NM_002834.5(*PTPN11*):c.5 C > T (U043) has increased coverage from 8× (singleton) to >20× (in trios sample) and NM_006929.5(*SKIV2L*):c.1404-2 A > G (U045) has increased coverage from 5× (singleton) to >30× (in trios sample). Due to the low coverage in these regions in the initial exome, the variants were likely filtered in the initial analysis and thus we were unable to reach a diagnosis. Also, improved bioinformatics by new copy number variants (CNVs) caller has allowed the detection of a heterozygous multi-exon deletion in individual U075 (in-frame deletion of *SPTAN1* exons 10–12), who presented with developmental delay and hypotonia. Individuals with large in-frame deletions located outside of the heterodimer domain have been reported to have milder phenotypes (varying from developmental delay with or without epilepsy to epileptic encephalopathy)^41^.

Lastly, the diagnosis of individual U036 was confirmed through international collaboration for new syndrome discovery: *MN1* C-terminal truncation syndrome, where 23 individuals who harbor truncating variants at C-terminal of *MN1* gene were found to share strikingly similar neurodevelopmental and craniofacial features^51^. It is postulated that these C-terminal truncating variants have escaped nonsense-mediated mRNA decay, thus creating a gain-of-function effect that increased protein stability, diminished cell proliferation, and enhanced *MN1* aggregation^51,52^.

### Diagnostic ES resulted in change in clinical management, outcome, and healthcare costs

We clinically followed up 36 individuals with diagnoses made in the initial study. The median length of follow-up was 1255 days (3.4 years; mean = 1223.6 days; range: 72–2250 days). Families who only came for second opinion/diagnostic purpose (no other consultation record in Hong Kong public hospitals; *n* = 5; U001, U039, U055, U081, and U100), proband who passed away before the diagnosis was made (*n* = 1; U049), and those who refused to receive the genetic result (*n* = 1; U017) were excluded. The survival rate is 97% at the time of this publication as one individual (U023) passed away during this period (follow-up length of 72 days).

A change in clinical management was observed in 72.2% of the families (26/36) that are comparable to the 84% (36/43) prediction by Mak et al. at the time of molecular diagnosis^27^. Table 2 showed the actual change in clinical management on the six major types of interventions in accordance to Riggs et al.^53^. The change in management led to a positive impact in the clinical outcome in four individuals (11%). The costs associated with these changes in clinical management and outcomes are presented in Table 3. The diagnosis of Fanconi anemia for infant U005 has facilitated timely bone marrow transplantation, using the cord blood from his unaffected, HLA-matched sibling. This transplantation avoided life-long blood transfusions, and led to surveillance for non-hemic malignancy. In individual U090, the diagnosis of Costello syndrome in a child with unexplained failure-to-thrive allowed early detection of hypertrophic cardiomyopathy, which is a possibly fatal complication of the syndrome. In contrast to other myasthenic syndrome patients where acetylcholinesterase inhibitors were usually used as the treatment, in individual U092, the genetic diagnosis of myasthenic syndrome type 8 allowed genotype-directed therapy. Acetylcholinesterase inhibitors such as Mestinon (pyridostigamine) were found to have negative response in this specific subtype^54^. Instead, Ventolin (salbutamol), a common drug for treating asthma, has better treatment response and prevented individual U092 from recurrent respiratory infection and ICU admissions. Lastly, individual U102 has undergone pallidal deep brain stimulation, a surgical procedure commonly used for treating Parkinsonism, to stabilize his progressive dystonia that was shown to be an effective treatment for patients with the genetic defect in *KMT2B*^55^. The patient had good response to the treatment and showed improvements in speech, head extension, and increased voluntary movement in lower limbs.

**Table 2:** Longitudinal follow-up on the actual change in clinical management in 36 diagnosed patients in initial study.

ID	Diagnosis (OMIM)	Gene	Length of follow-up (days)		Change in management	R	D	P	S	L	M
U003	Alpha-thalassemia/mental retardation syndrome, X-linked (#301040)	*ATRX*	2070		Yes	√	√		√		
U004	Epilepsy, focal, with speech disorder with or without mental retardation (#245570)	*GRIN2A*	1882		Yes		√				√
U005	Fanconi anemia, complementation group A (#227650)	*FANCA*	1927		Yes			√	√		
U006	Schuss-Hoeijmakers sydnrome (#615009)	*PACS1*	2250		Yes		√				
U010	Axenfeld-Rieger syndrome, type 3 (#602482)	*FOXC1*	854		Yes				√		
U012	10q26 microdeletion syndrome (#609623)	10q26.2-qter deletion	1304		Yes		√				
U015	Coffin-Siris syndrome 1 (#135900)	*ARID1B*	1350		Yes	√					
U023	Beta-ueridopropionase deficiency (#613161)	*UPB1*	72		No						
U025	Bainbridge-Ropers syndrome (#615485)	*ASXL3*	197		No						
U027	Shwachman-Bodian-Diamond syndrome (#260400)	*SBDS*	1731		Yes	√			√	√	√
U028	Coenzyme Q_10_ deficiency, primary, 7 (#616276)	*COQ4*	245		Yes						√
U030	Coffin-Siris syndrome 2 (#614607)	*ARID1A*	1699		Yes		√		√		
U031	Hyperphosphatasia with mental retardation syndrome (HMRS) 2 (#614730)	*PIGO*	1451		No						
U033	X-linked congenital disorder of glycosylation type Iim (#300896)	*SLC35A2*	1684		No						
U040	Bainbridge-Ropers syndrome (#615485)	*ASXL3*	1589		No						
U042	Early infantile epileptic encephalopathy (#612164)	*STXBP1*	1632		Yes				√		√
U044	Noonan syndrome-like disorder with loose anagen hair 2 (#617506)	*PPP1CB*	1211		Yes		√		√		
U050	Lenz-Majewski hyperostotic dwarfism (LMHD) (#151050)	*PTDSS1*	1507		Yes	√	√		√		
U052	Autosomal recessive agenesis of the corpus callosum with peripheral neuropathy (#218000)	*SLC12A6*	1282		Yes				√		
U062	Neurodevelopmental disorder a.o. Rett syndrome (#312750)	*MECP2*	1393		No						
U063	Congenital megaconial muscular dystrophy (#602541)	*CHKB*	1519		Yes		√		√		
U068	Okur-Chung neurodevelopmental syndrome (#617062)	*CSNK2A1*	272		No						
U069	Mental retardation, autosomal dominant 31 (#616158)	*PURA*	414		No						
U074	Pallister-Killian syndrome (#601803)	12p13.33-p11.1 duplication	988		Yes	√					
U076	Keratitis-ichthyosis-deafness syndrome (#148210)	*GJB2*	1384		Yes	√			√		
U080	Mental retardation, X-linked 102 (#300958)	*DDX3X*	1248		No						
U083	X-linked dominant Neurodegeneration with Brain Iron Accumulation 5, NBIA5 (#300894)	*WDR45*	1151		Yes		√		√		
U084	Spinal muscular atrophy, lower extremity-predominant 1, AD (#158600)	*DYNC1H1*	1217		Yes		√				
U087	Auriculocondylar syndrome 1 (#602483)	*GNAI3*	1194		No						
U089	Insensitivity to pain, congenital, with anhidrosis (#256800)	*NTRK1*	1251		Yes		√		√		
U090	Costello syndrome (#218040)	*HRAS*	1259		Yes	√	√		√		√
U092	Myasthenic syndrome, congenital, 8, with pre- and postsynaptic defects (#615120)	*AGRN*	1075		Yes	√			√		√
U098	Charcot-Marie-Tooth disease, axonal, type 2 S (#616155)	*IGHMBP2*	934		Yes					√	
U099	Nicolaides-Baraitser syndrome (#601358)	*SMARCA2*	1081		Yes				√		
U102	Dystonia 28, childhood-onset (#617284)	*KMT2B*	1039		Yes	√		√			
U103	Aarskog-Scott syndrome (#305400)	*FGD1*	693		Yes		√		√		
		Median length:	1255	Count:	26	9	13	2	17	2	6
				Percentage:	72.2%	25%	36%	6%	47%	6%	17%

*R* Referral to specialist, *D* Diagnostic testing, *P* Procedure, *S* Surveillance, *L* Lifestyle changes, *M* Medication.

**Table 3:** Patients with changes in clinical outcomes and the associated healthcare costs.

ID	Gene	Diagnosis (OMIM)	Expected outcome without treatment	Change in management	Change in outcome	Additional cost (HKD; 1st year)	Avoided cost (HKD; 1st year)	Cost difference (HKD; 1st year)
U005	*FANCA*	Fanconi anemia, complementation group A (#227650)	1. Clinical management as per clinical suspicion of Pearson syndrome 2. Continued need for blood transfusions	1. Bone marrow transplantation using cord blood from non-affected, matched sibling ($1,094,396) 2. Follow-up in oncology clinic for surveillance for non-hemic malignancy; two appointments in the past 5 years; $492 pa) 3. Avoided further blood transfusions (nine blood transfusions in the previous 7 months; $111,384 pa)	Curative, no further blood transfusions	$1,094,888 (USD$140,370; €124,419)	$111,384 (USD$14,280; €12,657)	$983,504 (USD$126,090; €117,762)
U090	*HRAS*	Costello syndrome (#218040)	1. Progressive failure to thrive; severe progressive HCM due to delay in treatment	1. Follow-up in cardiology clinic (two appointments per year; $2,460 pa) 2. Follow-up in hematology clinic (one appointment per year; $1,230 pa) 3. Follow-up in oncology clinic (one appointment per year; $1,230 pa)	Early detection of HCM	$4,920 (USD$631; €559)	0	$4,920 (USD$631; €559)
U092	*AGRN*	Myasthenic syndrome, congenital, 8, with pre- and postsynaptic defects (#615120)	1. Recurrent hospital admissions due to respiratory infection 2. Inappropriate use of acetylcholinesterase inhibitor (Mestinon (pyridostigamine)) for other Myasthenic syndrome subtypes	1. Initiated Ventolin (salbutamol) 4 mg tds ($218 pa) 2. Avoided Mestinon (pyridostigamine; minimum of $274 pa) 3. Avoided further ICU admissions (three ICU admissions in the previous 3 years, average of 3 days each; $46,050 pa)	No further ICU hospital admissions; reduced fatigability	$218 (USD$28; €25)	$46,324 (USD$5939; €5264)	−$46,106 (−USD$5911; −€5239)
U102	*KMT2B*	Dystonia 28, childhood-onset (#617284)	1. Progressive deterioration of dystonia 2. Recurrent bilateral patellar dislocation 3. Tendon transfer surgery 4. Wheelchair bound	1. Pallidal deep brain stimulation ($85,262) 2. Avoided tendon transfer surgery ($36,516)	Stabilized	$85,262 (USD$10,931; €9,689)	$36,516 (USD$4681; €4150)	$48,746 (USD$6249; €5539)
Total cost difference:								$991,064 (USD$147,059; €112,621)

*HCM* hypertrophic cardiomyopathy, *ICU* intensive care unit, *pa* per annum, *tds* three times daily.

### Diagnostic ES influenced parents’ reproductive decision

A questionnaire on the reproductive planning and decision was distributed to each parent of initial ES-positive individuals. A total of 36 questionnaires (19 fathers and 17 mothers from 22 families) were completed and returned with a response rate of 59%. The marital status was indicated as ‘married’ in 19 families, ‘remarried’ in two, and ‘divorced’ in one family. Of the 36 respondents, 61% (22/36) agreed that the genetic diagnosis affected his/her decision to have more biological children.

Regarding the use of prenatal diagnosis (PND) and preimplantation genetic diagnosis (PGD), of all the respondents, 78% (28/36) thought PND should be offered to families with the same genetic diagnosis and 75% (27/36) would consider using PND for themselves. Interestingly, 56% (19/36) of the respondents agreed PGD should be offered to families with the same genetic diagnosis, but only 36% (13/36) of them would consider PGD at their personal level. In fact, most of the respondents (47%; 17/36) would not consider PGD for themselves.

**Fig. 1:**
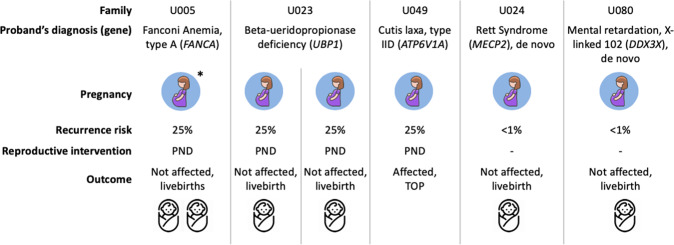
The pregnancy outcomes of five families who sought advice in assisted reproductive service within the study’s follow-up period (median of 3.4 years). *PND* prenatal diagnosis, *TOP* termination of pregnancy; *: ongoing twin pregnancy at the time of genetic diagnosis.

The reproductive outcome of the 37 families with diagnostic ES were collected (including the family with proband passed away before genetic diagnosis), using the electronic patient record. Five couples had sought advice from reproductive services (Fig. 1). There were a total of six pregnancies over this follow-up period from these five families. Family U023 had two pregnancies. Two couples (parents of U062 and U080) did not receive any reproductive intervention after consultation as the recurrence risk is low (de novo variant). Three couples (four pregnancies) underwent PND for autosomal recessive conditions. Of note, mother of U005 was pregnant with a pair of twins at the time of diagnosis, therefore, only PND could be offered. One of the four pregnancies was affected and resulted in termination of pregnancy. None of the family underwent PGD in this cohort.

## Discussion

**Fig. 2:**
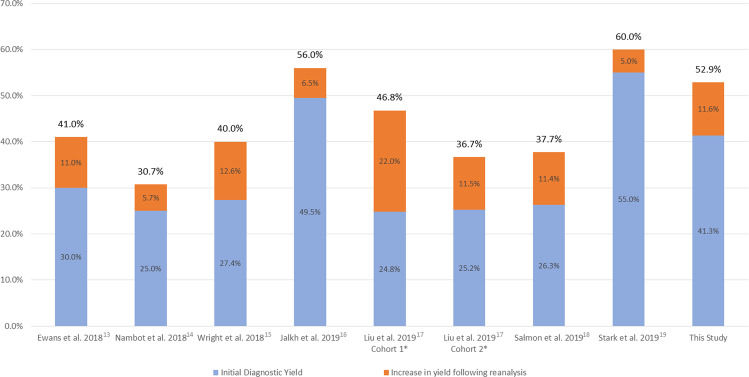
The diagnostic yield of exome sequencing initial analysis and reanalysis from published studies and our current study. We searched PubMed with the search terms “exome” and “reanalysis” to identify relevant studies. There were no language and date restrictions. Only studies that provide both the initial analysis and reanalysis data were included. *Liu et al. cohort 1 and Liu et al. cohort 2 are two separate patient cohorts from the same reanalysis study with different approach and time frame (cohort 1: manual reanalysis over a 5-year period; cohort 2: semiautomated reanalysis over a 4-year period)^17^.

This study demonstrated the diagnostic capacity of ES reanalysis for patients suspected of genetic diseases. Twelve additional diagnoses were made by ES reanalysis in 46 individuals at a time frame of 3.2 years after the initial analysis. The diagnostic rate within the reanalysis group is 26% (12/46), while the additional yield compared with the initial analysis is at least 12% (increased yield from 41% to at least 53%). The diagnostic yield in this study showed comparable results to other published studies with reanalysis diagnostic yield of 5–36%; and additional diagnostic yield of 5–22% at 6–36 months after initial analysis (Fig. 2)^10–23^.

A higher additional diagnostic yield would be expected if all families participated in the reanalysis (13 could not be contacted and two refused to participate). There was difficulty in recontacting all families as some of the patients are from mainland China and Taiwan. One of the families refused to participate because the child has been improving so they do not think there is a need for reanalysis.

Among the 46 negative cases reviewed, three individuals (U022, U077, and U104) received a genetic diagnosis through genetic evaluations in other centers during this period, either via the clinical or research route. No diagnosis was made by routine nongenetic clinical evaluation. One individual participated in another ES research, in which the diagnosis made was consistent with our finding (U077; NM_001130438.3(*SPTAN1*):c.4828 C > T). Two individuals had the diagnoses through standard of care genetic testing available in the Hong Kong health system. U022 received the same genetic diagnosis of *PACS1* variant through NGS panel testing. U104 had a clinical suspicion of myotonic dystrophy on reassessment and therefore targeted testing was performed. It was found that she has expanded repeats (>667) in *DMPK*, confirming the diagnosis. The diagnosis of U104 was not detectable by ES reanalysis as the repeat expansion is located in 3′ untranslated region of *DMPK*, which would not be captured by a typical exome. Furthermore, short read sequencing from ES/GS has technical limitations in detecting these expanded repeats that specialized tools have modest success in detecting expansion outliers^56,57^. By comparing the additional yield, ES reanalysis remains as a strong candidate to tackle the “experimental maze” after negative exome result. However, clinicians should be aware of its limitation and consider other possible approaches.

Currently, GS is viewed to be the ultimate genetic diagnostic test to detect variations in the genome. Therefore, after negative exome, clinicians often consider GS to be one of the possible solutions. In fact, ES reanalysis is recommended before GS due to GS’s relatively high cost yet low diagnostic rate^21^. To date, there is a lack of back-to-back comparison between the diagnostic capacity of ES reanalysis and GS. Alfare et al. proceeded to GS following negative ES, they postulated that 30% of the new findings should be detectable by ES reanalysis, and therefore GS could only achieve 7% higher detection rate than ES reanalysis, further supporting the role of ES reanalysis in clinical setting for patients with suspected genetic disease^58^.

This study presented a variety of reasons for the improved diagnostic yield: strengthened gene–disease association and literature updates (50%), additional information on patient phenotypes (33%), additional sequencing for trios/other affected family members (25%), improvement in sequencing data by re-sequencing (17%), improvement in bioinformatics tools (8%), as well as research collaborations through international case sharing platforms (8%; Table 1). This reinforces the need for reanalysis, as with time, new gene– and variant–disease associations may be discovered, patients may develop new phenotypes, in combination with advancing sequencing and bioinformatic technology, could contribute to an increased diagnostic yield of ES. Although there is no marked difference in the detection rate by reanalysis of raw data (4/11, 36.3%) and re-sequencing (8/35, 22.8%), the result has illustrated that re-sequencing to generate new exome data could be critical for additional diagnosis. Two diagnoses were achieved due to the increase in coverage in region containing the variant (U043 and U045), it could be missed if only data reanalysis has been performed. Therefore, re-sequencing may have an advantage of better capturing the variant as compared to data reanalysis. However, since the initial ES of this study was performed in commercial laboratories, we do not have access to all data for parallel comparison of the performance in reanalysis and re-sequencing. More systematic study should be conducted to evaluate and compare the diagnostic capacity, as well as the cost-effectiveness of keeping data for reanalysis versus keeping DNA for re-sequencing.

In this cohort of 104 pediatric patients, two patients have the same diagnosis of Schuss-Hoeijmakers syndrome; U006 was diagnosed in the initial ES in 2013 (trios), while U022 was diagnosed in the reanalysis phase (singleton). Reviewing the clinical history of the two patients in our cohort, it was surprising to find that U022’s initial negative ES was performed in 2014, later than the diagnosis of U006. The initial ES for these two cases were performed in different laboratories with different sequencing and analytical methodologies. Although from a diagnostic point of view, missing a diagnosis is not ideal, this reflected the difficult reality. Apart from the sequencing and variant calling capacity, laboratories have different analytical pipelines, and therefore may have different interpretation toward the new and limited evidence, especially before the publication of ACMG guidelines on variant interpretation in 2015^59^. During this rapidly evolving era of genomics where new genes and its disease association are discovered, genetic diagnosis and variant classification are dynamic and changeable over time, stressing the importance of data reanalysis. Also, this example illustrated the benefits of having trio samples. It allowed the possible detection of de novo variants, which was the key to reaching the diagnosis in U006. The use of the “de novo” paradigm is known to be important in the analysis of certain diseases and variant types, such as in neurodevelopmental disorders. A meta-analysis has shown that the odds of reaching diagnosis with a trio design is double that of singleton^5^. Within this reanalysis cohort, samples from 20 individuals were reanalyzed with additional family members (19 trios and 1 duo with affected sibling), while 26 were singletons. The diagnostic yield from trio samples (6/19, 31.5%) was higher than that of singleton samples (5/26, 19.2%). Therefore, we recommend trio analysis for maximizing the diagnostic utility of ES. However, if there is a resource constraint, a stepped approach should be adopted, starting with singleton ES and if negative, proceed to trio sequencing during reanalysis.

To date, this is the third longitudinal follow-up study, with the longest study duration, on investigating the long-term benefit of ES diagnosis on patient care and outcome. Over the 3-year follow-up period, 26 out of 36 diagnosed individuals (72%) have implemented changes in their management as a result of ES diagnosis, of which four (11%) had a long-term change in clinical outcome, consequently saving costs from the healthcare system perspective in the long term. This is comparable to a similar study by Stark et al., which reported at over a year of follow-up, 16 out of 48 diagnosed infants had a change in management, and among these, four had a change in clinical outcome or hospital service use^19^.

At the initial report of this cohort, changes in management were recommended by clinical geneticists based on disease-specific management guidelines, case reports, or known function of genes of the diagnosed patient. From the result of the longitudinal follow-up, the change in clinical management is generally in line with the prediction. However, there are slight discrepancies, especially on the aspects of specialist referrals (predicted = 44%, actual = 25%), lifestyle change (predicted = 14%, actual = 6%), and medication (predicted = 28%, actual = 17%). This discrepancy might be attributed to case-specific applicability of the advised changes (e.g., some patients have already developed complications prior to genetic diagnosis and therefore already seeing specialists or taking appropriate medications due to the presenting symptoms), ineffective communication (genetic diagnosis was not communicated to other responsible specialists in some cases), and clinicians’ perceived importance of the ES results (some clinicians did not make management changes based on the genetic diagnosis provided by geneticists). Furthermore, lifestyle changes would be difficult to measure in a clinical setting and may not be recorded in the medical consultations. For example, sun protection is recommended for individual U076 with keratitis-ichthyosis-deafness syndrome. The inability to measure whether certain lifestyle change has been implemented would be a limitation to this study. The discrepancy also demonstrated the importance of long-term follow-up data, as acute changes in management might not necessarily be followed through over the clinical course of patients. Further exploring the factors affecting the delivery and actionability of ES results in the clinical setting could be important to maximize the value of diagnostic ES.

Consistent with the initial prediction, ES mostly assisted the clinical management in the aspects of surveillance for potential complications (47%, 17/36), further diagnostic testing (36%, 13/36), and specialist referrals (25%, 9/36) within the individual health domain. This is also in line with Niguidula et al., which demonstrated majority of patients benefitted from a positive ES diagnosis in the areas of prognosis and enhanced surveillance^29^. Indeed, it is also the clinicians’ perception that impact on prognostication and disease surveillance are some of the most important outcomes of a positive exome result^60^. In contrast to the individuals reported by Stark et al.^19^, where changes in health outcome were all resulted from changes in medication or procedural interventions, one individual (U090) in our cohort had a significant change in health outcome due to implementation of surveillance. With the diagnosis of Costello syndrome, cardiac surveillance by echocardiogram was initiated as cardiomyopathy is a known complication of the syndrome. In fact, early asymmetrical hypertrophic cardiomyopathy was detected 2 months after the disclosure of genetic result. It could be otherwise unnoticed and likely fatal until symptoms present. The genetic diagnosis has led to the prevention insidious life-threatening event of cardiomyopathy.

The genetic diagnosis did not change the clinical management in ten individuals. Nine of them have intellectual disability syndrome, in which the developmental support and management plan do not fall into one of the six categories. For individual U087 with the diagnosis of Auriculocondylar syndrome, no new treatment was initiated, but we connected the family to the surgical expert in doing surgery for this syndrome. U087 is now in joint care by local hospital and expert surgeons from Taiwan.

A diagnostic genetic test does not only benefit the clinical management and outcome, it also provides information that aids reproduction and family planning. By knowing the mode of inheritance and carrier status, the reproductive risk of parents can be determined and couple can make use of the information to decide on the reproductive plan. From our survey result, the confidence in the use of PND is considerably higher than that of PGD. Over 70% of the respondents agree that PND should be offered to families with the same genetic diagnosis, and they would consider PND if they are planning for future pregnancies. Only 56% of them agree PGD should be offered and even less (36%) would consider it for themselves. Looking into the actual pregnancy outcomes, in fact, none of the families chose PGD as the alternative assisted reproduction option. Here, we outline several reasons for such. Firstly, among the surveyed families with inheritance determined (*n* = 21), the majority (76%, 16/21) have a recurrence risk of <1% as the variant identified in the proband is a de novo or mosaic variant. Usually, PGD would be more attractive to couples with higher recurrence risks. In these 21 families, only 24% (5/21) of them has a 25% recurrence risk (parents being a carrier of an autosomal or X-linked recessive disease) and none of them has an inherited autosomal dominant disorder. Secondly, the proactiveness of having more babies. PGD is a more active intervention that the couple needs to decide and seek assistance before being pregnant, while PND may be accepted as a test or a solution after getting pregnant. Having an ill child may have created struggle in the family that would hinder the wanting of another child despite PGD could significantly reduce the risk, as 61% of the respondents reflected that the genetic diagnosis affected their decision to have more children. Similarly, van Rij et al. observed a negative association between the presence of a living, affected child and PGD intention (OR 0.5, 95% CI 0.3–1.1), and use (OR 0.3, 95% CI 0.1–0.6) in 246 couples with high reproductive risk of a range of chromosomal or Mendelian disorders^61^. On the other hand, a study from Hong Kong in 2002 showed parents with children affected with homozygous beta thalassemia showed more positive attitude toward the use of PGD than PND as an alternative^62^. This may be due to the potential advantage of selecting HLA-matched embryos for the affected child. Thirdly, couples may take into consideration that the PGD-IVF ongoing pregnancy rate in Hong Kong is only 33.1%^63^. Also, there is a maternal age requirement, PGD request will not be accepted if the maternal age is over 40 years old in Hong Kong. Given people are getting married and giving birth at a later age in modern society like Hong Kong, the applicability and feasibility of PGD are limited even if the couples want to have another child. The median age of mothers filling in the questionnaire was 41 (mean = 41.8; range = 33–52) years old. Lastly, couples have to bear the cost of PGD-IVF themselves as it is not subsidized in the Hong Kong healthcare system. The high cost of at least HKD$40,000 (United State dollars (USD)$5128; €4545) per cycle would be another factor that prevented couples from choosing PGD. Two couples who had sought advice from assisted reproductive service expressed their concern on the financial cost of PGD, and eventually decided not to proceed with PGD.

From a healthcare system standpoint, unnecessary hospital admissions, management procedures, and treatment medications could be avoided as a result of the genetic diagnosis. Four individuals (11%) had a long-term change in clinical outcome; of which changes in routine management, such as avoidance of routine procedures (i.e., blood transfusions), avoidance of frequent hospital admissions, and initiation of routine clinical follow-ups have led to a minimum healthcare cost saving of HKD$152,078 (USD$19,497; €17,282) annually for these four individuals. Further cost savings would be seen in a longer term, and levelled off in 6.5 years for these four individuals, as total cost of certain operations, such as bone marrow transplantation and deep brain stimulation would have been incurred as a one-off cost, with minimal follow-up costs in the following years. In Stark et al.’s cohort, it was found to save $1578 Australian dollars (HKD$8456) per quality-adjusted life year (QALY) gained at 1-year-follow-up^19^. Although a comprehensive cost-utility analysis is not performed in our study as QALY was not collected, it could be anticipated that the positive outcome of additional life span, quality of life gained, lifetime value of unaffected children through assisted reproductive methods, etc. would lead to clear dominance (better outcome at a lower cost) of ES from the individual, family, healthcare system, and societal perspective.

To conclude, this 3-year follow-up study demonstrated the diagnostic and clinical utility of ES and reanalysis in a cohort of 104 pediatric individuals. ES reanalysis is a useful tool that provides promising additional diagnoses compared to routine clinical evaluation. ES and subsequent periodic reanalysis are recommended in undiagnosed individuals as genetic knowledge and literature is regularly updated. This study also provided quantification to the long-term clinical utility of genetic diagnoses, which has a positive impact on individual’s clinical management, parents’ reproductive planning, and healthcare cost savings in a long run. Our study highlights the benefits of implementing ES and regular reanalysis in the clinical setting.

## Methods

### Cohort characteristics

This is a follow-up analysis to our previous study, Mak et al., where 104 individuals with suspected genetic disorder were recruited for ES in 2013–2017^27^. ES was performed in one of the two clinical commercial laboratories, 95 by Genome Diagnostic Nijmegen and nine by Ambry Genetics. A total of 78% (81/104) of the individuals were analyzed as singletons, followed by targeted Sanger sequencing of parental samples in HKU if candidate variant was identified. The remaining 23 individuals with ES were analyzed as trios. Detailed sequencing methodology and variant interpretation approach of the initial ES were described in the original paper by Mak et al.^27^. The paper concluded a diagnostic yield of 41% (43/104 received a genetic diagnosis) and predicted that 84% of them would result in a change in clinical management.

### Exome reanalysis: patient recruitment and data processing

Individuals without a diagnosis in the initial ES study (*n* = 61) were recruited for reanalysis, in collaboration with Yale University. The median time frame from the initial analysis to reanalysis was 1178.5 days (3.2 years; mean: 1150 days; range: 301–1039 days). Families that were unable to be contacted or refused consent for reanalysis were excluded. Depending on the availability of raw data, either raw sequencing data were reanalyzed, or stored DNA were re-sequenced and analyzed. For re-sequencing, the IDT xGen Exome Panel exome capture kit was used and 100 bp paired-end reads sequencing was performed on the Illumina NovaSeq 6000 platform. Reads were aligned to hg19 reference genome using bwa-mem and processed following GATK best practice guidelines, including variant calling with HaplotypeCaller^64–67^. CNVs were called using gcnv (part of GATK4). The exact data processing and variant calling pipeline can be accessed at https://github.com/leklab/cromwell_wdl/tree/master/gatk4_multisample. The variants were annotated using Variant Effect Predictor through Hail (https://github.com/hail-is/hail) and then uploaded to seqr (https://github.com/macarthur-lab/seqr) for analysis^68^. For CNV analysis, variant calls from gcnv were annotated using AnnotSV^69^.

### Exome reanalysis: data interpretation

Using seqr interface, only rare coding and splice-site variants (allele frequency <1% in gnomAD genomes, gnomAD exomes, ExAC, 1000 genomes, and TOPMED bravo databases) were included in downstream analysis. We filtered variants with a genotype quality < 20, alternate allele balance <25%, and non-PASSing variants sites determined by variant quality score recalibration. Missense, in-frame, frameshift, nonsense, and essential splice site variants are included in downstream analysis; synonymous and intronic variants were included only when there was a strong suspicion in a candidate gene.

For trio exomes, de novo and biallelic rare variants were prioritized. For singletons, genes with heterozygous variants absent from gnomAD, rare homozygous variants or two rare heterozygous variants were selected to match with individual’s clinical phenotype. A diagnosis is reached when the selected variant could be classified as pathogenic/likely pathogenic by the ACMG guidelines^59^. Sanger sequencing was performed to confirm the finding and segregation studies were undertaken if possible.

If a CNV overlapped with coding regions of a gene that had been associated with a phenotype matching with the individual’s clinical features, it was considered for disease causality. CNVs were validated by Seqplexing (Valencia, Spain) using EOSAL-CNV (Easy One-Step Amplification and Labeling procedure for CNV detection), which utilized a tailed primer with fluorescent probe for amplification of samples and control^70^. Amplified products were then loaded onto a capillary DNA sequencer for sizing and quantification. Analysis was done by comparing results of the samples and controls.

### Long-term follow-up: clinical outcome

For individuals with an earlier diagnosis from initial ES (*n* = 43), we measured the long-term clinical utility by clinical follow-up and review of their medical records. The length of follow-up was measured from the date of result disclosure to the latest follow-up date in the electronic patient health record. We evaluated whether the genetic diagnosis has led to a change in individuals’ clinical management and/or outcome in accordance to the six aspects suggested by Riggs et al., in which we have adopted this classification in our initial study: referral, diagnostic testing, procedure, surveillance, lifestyle changes, and medication^27,53^.

### Long-term follow-up: cost analysis

For individuals with changes in management and clinical outcomes as a result of the diagnosis, we have estimated the costs of investigations, treatment that were initiated and/or avoided, and healthcare service utilization. Costs were obtained from the Hong Kong Hospital Authority. For procedural costs that were not publicly available in the Hong Kong Hospital Authority setting, costs were obtained using the United Kingdom National Health Service National schedule of reference costs^71^. All costs in this study are reported in Hong Kong dollars, with an exchange rate of ~7.8 per USD and 8.8 per European dollar (Euro) at the time of study.

### Long-term follow-up: reproductive outcome

Information related to parents’ use and views on assisted reproductive service and their pregnancy outcome were collected. There are two major options in assisting families with genetic conditions: PGD and PND. A questionnaire on the opinion toward these options was set out: whether PGD and PND should be offered to families with the same genetic diagnosis, and whether they would consider undergoing PGD and PND themselves. The questionnaire was distributed to each parent during their child’s medical follow-up or mailed to them for completion. Electronic patient record was reviewed to collect information on actual service use on these reproductive methods.

### Ethics approval

Ethics approval was granted by the Institutional Review Board, the University of Hong Kong/Hospital Authority Hong Kong West Cluster (UW 12–211). Written informed consents have been obtained from the parents of the participants.

### Reporting summary

Further information on experimental design is available in the Nature Research Reporting Summary linked to this paper.

## Supplementary information

Supplementary file

Reporting Summary Checklist FLAT

## Data Availability

Sequencing and phenotype data that support the findings of this study have been deposited in dbGaP and AnVIL with the accession code phs000744.

## Code availability

Bioinformatics codes used to process the data in this study are deposited in GitHub with no access restriction, details are provided in the “Methods” section.
