# Supplementary material for: A three-year follow-up study evaluating clinical utility of exome sequencing and diagnostic potential of reanalysis
Source: NPJ Genom Med. 2020 Sep 10;5:37. doi: 10.1038/s41525-020-00144-x (PMC7484757; doi:10.1038/s41525-020-00144-x)
Supplement: Supplementary file 1 — Supplementary file [file 41525_2020_144_MOESM1_ESM.pdf]

Supplementary Table 1. Data source and reanalysis approach to the 46 participated families

ID	Initial WES approach	Reanalysis data source	Reanalysis approach	Time between initial WES and reanalysis (days)
U002	Trios	DNA	Singleton	1709
U008	Trios	Existing raw data	Trios	1739
U009	Singleton	DNA	Singleton	1551
U011	Trios	DNA	Singleton	1535
U013	Singleton	DNA	Singleton	1454
U014	Trios	Existing raw data	Singleton	1189
U018	Singleton	DNA	Trios	1389
U019	Singleton	DNA	Trios	713
U020	Singleton	DNA	Trios	1411
U021	Singleton	DNA	Trios	1469
U022	Singleton	DNA	Singleton	1512
U024	Singleton	DNA	Trios	929
U026	Singleton	DNA	Trios	1384
U029	Singleton	DNA	Trios	1213
U036	Singleton	Existing raw data	Trios	301
U037	Singleton	Existing raw data	Singleton	1428
U038	Singleton	DNA	Trios	1327
U043	Singleton	DNA	Trios	1231
U045	Singleton	DNA	Trios	1306
U046	Singleton	DNA	Singleton	1365
U048	Singleton	DNA	Singleton	1384
U051	Singleton	DNA	Trios	1331
U054	Singleton	DNA	Singleton	1278
U057	Singleton	Existing raw data	Duo	939
U058	Singleton	DNA	Singleton	530
U060	Singleton	DNA	Trios	1218
U061	Singleton	DNA	Trios	1168
U065	Singleton	DNA	Singleton	1098
U066	Singleton	DNA	Singleton	1055
U067	Singleton	DNA	Singleton	1197
U070	Singleton	DNA	Singleton	1036
U071	Singleton	DNA	Singleton	1372
U072	Singleton	DNA	Trios	1035
U075	Singleton	DNA	Singleton	1131
U077	Singleton	Existing raw data	Singleton	851
U078	Singleton	DNA	Singleton	965
U079	Singleton	DNA	Trios	936
U082	Singleton	Existing raw data	Singleton	957
U085	Singleton	Existing raw data	Singleton	1092
U086	Singleton	Existing raw data	Singleton	1073
U088	Singleton	Existing raw data	Singleton	1148
U091	Singleton	DNA	Singleton	917
U093	Singleton	Existing raw data	Singleton	934
U094	Trios	DNA	Trios	646
U101	Singleton	DNA	Trios	770
U104	Singleton	DNA	Singleton	678

Supplementary Table 2. Variants' pathogenicity classification according to the ACMG guidelines 2015 and the submitted ClinVar accession

ID	Gene	Variant	Zygosity	Segregation	Pathogenicity according to ACMG guidelines 2015	Classification Criteria	ClinVar Accession
U018	*ATP1A3*	NC_000019.9:g.42489109G>C<br>NM_152296.5:c.954C>G, p.(Ile318Met)	Heterozygous	*De novo*	Pathogenic	PS2, PM1, PM2, PP2, PP3	SCV001364379
U022	*PACS1*	NC_000011.9:g.65978677C>T<br>NM_018026.4:c.607C>T, p.(Arg203Trp)	Heterozygous	*De novo*	Pathogenic	PS2, PS4, PM1, PM2, PP3	SCV001364380
U036	*MN1*	NC_000022.10:g.28146988_28146997dup<br>NM_002430.3:c.3870_3879dupTGACGCCAAG,<br>p.(Ala1294Ter)	Heterozygous	*De novo*	Pathogenic	PVS1, PS2, PS3, PM2	VCV000812561.1
U043	*PTPN11*	NC_000012.11:g.112856920C>T<br>NM_002834.5:c.5C>T, p.(Thr2Ile)	Heterozygous	*De novo*	Pathogenic	PS2, PS4, PM2, PP2, PP3	SCV001364381
U045	*SKIV2L*	NC_000006.11:g.31931188A>G<br>NM_006929.5:c.1404-2A>G	Heterozygous	Paternal	Likely Pathogenic	PVS1, PM2	SCV001364382
		NC_000006.11:g.31931511G>A<br>NM_016138.5:c.1647+1G>A	Heterozygous	Maternal	Likely Pathogenic	PVS1, PM2	SCV001364383
U057	*PRF1*	NC_000010.10:g.72358459C>T<br>NM_005041.5:c.1018G>A, p.(Asp340Asn)	Homozygous	Inherited from parents	Likely Pathogenic	PS3, PM2, PP1, PP4, PP5	SCV001364384
U066	*MFN2*	NC_000001.10:g.12058934C>T<br>NM_001127660.1:c.707C>T, p.(Thr236Met)	Heterozygous	Unknown	Likely Pathogenic	PM1, PM2, PP2, PP3	SCV001364385
U071	*COL11A1*	NC_000001.10:g.103427475C>T<br>NM_001854.4:c.3115G>A, p.(Gly1039Ser)	Heterozygous	Unknown	Likely Pathogenic	PM1, PM2, PP2, PP3	SCV001364386
U075	*SPTAN1*	NC_000009.11:g.(?_131341916)_(131344171_?)del<br>NM_001130438.3:c.(?_1225)_(1572_?)del<br>(exon 10-12 deletion)	Heterozygous	*De novo*	Pathogenic	PVS1, PS2, PM2	SCV001364387
U077	*SPTAN1*	NC_000009.11:g.131374047C>T<br>NM_001130438.3:c.4828C>T, p.(Arg1610Trp)	Heterozygous	*De novo*	Likely Pathogenic	PS2, PM2, PP5	SCV001364388
U086	*GNB1*	NC_000001.10:g.1737942A>G<br>NM_002074.5:c.239T>C, p.(Ile80Thr)	Heterozygous	Unknown	Pathogenic	PS3, PS4, PM1, PM2, PM5, PP2, PP3	SCV001364389

U094	*COQ7*	NC_000016.9:g.19089425AG>TAATGCATC NM_016138.5:c.599_600delinsTAATGCATC, p.(Lys200Ilefs*56)	Heterozygous	Paternal	Likely Pathogenic	PVS1, PM2	SCV001364390
		NC_000016.9:g.19085309C>T NM_016138.5:c.319C>T, p.(Arg107Trp)	Heterozygous	Maternal	Likely pathogenic	PS3, PM2, PM3, PP3	SCV001364391
