# Supplementary material for: A three-year follow-up study evaluating clinical utility of exome sequencing and diagnostic potential of reanalysis
Source: NPJ Genom Med. 2020 Sep 10;5:37. doi: 10.1038/s41525-020-00144-x (PMC7484757; doi:10.1038/s41525-020-00144-x)
Supplement: Supplementary file 2 — Reporting Summary Checklist FLAT [file 41525_2020_144_MOESM2_ESM.pdf]

nature research

Corresponding author(s): Brian HY Chung, Monkol Lek

Last updated by author(s): Aug 3, 2020

Reporting Summary

Nature Research wishes to improve the reproducibility of the work that we publish. This form provides structure for consistency and transparency in reporting. For further information on Nature Research policies, see our Editorial Policies and the Editorial Policy Checklist.

Statistics

For all statistical analyses, confirm that the following items are present in the figure legend, table legend, main text, or Methods section.

n/a	Confirmed	
☒	☐	The exact sample size (*n*) for each experimental group/condition, given as a discrete number and unit of measurement
☒	☐	A statement on whether measurements were taken from distinct samples or whether the same sample was measured repeatedly
☒	☐	The statistical test(s) used AND whether they are one- or two-sided<br>*Only common tests should be described solely by name; describe more complex techniques in the Methods section.*
☒	☐	A description of all covariates tested
☒	☐	A description of any assumptions or corrections, such as tests of normality and adjustment for multiple comparisons
☒	☐	A full description of the statistical parameters including central tendency (e.g. means) or other basic estimates (e.g. regression coefficient) AND variation (e.g. standard deviation) or associated estimates of uncertainty (e.g. confidence intervals)
☒	☐	For null hypothesis testing, the test statistic (e.g. *F*, *t*, *r*) with confidence intervals, effect sizes, degrees of freedom and *P* value noted<br>*Give P values as exact values whenever suitable.*
☒	☐	For Bayesian analysis, information on the choice of priors and Markov chain Monte Carlo settings
☒	☐	For hierarchical and complex designs, identification of the appropriate level for tests and full reporting of outcomes
☒	☐	Estimates of effect sizes (e.g. Cohen's *d*, Pearson's *r*), indicating how they were calculated

Our web collection on statistics for biologists contains articles on many of the points above.

Software and code

Policy information about availability of computer code

Data collection	No software was used in the data collection
Data analysis	Bioinformatics codes used to process the data in this study are deposited in GitHub with no access restriction, details are provided in the Methods section.<br>"Reads were aligned to hg19 reference genome using bwa-mem and processed following GATK best practice guidelines including variant calling with HaplotypeCaller. Copy-number variants (CNV) were called using gcnv (part of GATK4). The exact data processing and variant calling pipeline can be accessed at https://github.com/leklab/cromwell_wdl/tree/master/gatk4_multisample. The variants were annotated using Variant Effect Predictor through Hail (https://github.com/hail-is/hail) and then uploaded to seqr (https://github.com/macarthur-lab/seqr) for analysis. For CNV analysis, variant calls from gcnv were annotated using AnnotSV."

For manuscripts utilizing custom algorithms or software that are central to the research but not yet described in published literature, software must be made available to editors and reviewers. We strongly encourage code deposition in a community repository (e.g. GitHub). See the Nature Research guidelines for submitting code & software for further information.

Data

Policy information about availability of data

All manuscripts must include a data availability statement. This statement should provide the following information, where applicable:

- Accession codes, unique identifiers, or web links for publicly available datasets
- A list of figures that have associated raw data
- A description of any restrictions on data availability

Sequencing and phenotype data that support the findings of this study have been deposited in dbGaP and AnVIL with the accession code phs000744.

Field-specific reporting

Please select the one below that is the best fit for your research. If you are not sure, read the appropriate sections before making your selection.

☒ Life sciences ☐ Behavioural & social sciences ☐ Ecological, evolutionary & environmental sciences

For a reference copy of the document with all sections, see nature.com/documents/nr-reporting-summary-flat.pdf

Life sciences study design

All studies must disclose on these points even when the disclosure is negative.

Sample size	A cohort of 104 predominantly Chinese paediatric individuals from the previous publication PMID: 30109123 were recontacted for this follow-up study. No sample size calculation has been obtained as this is a follow-up study to the previous cohort.
Data exclusions	Individuals who are lost to follow-up or refused to participate in the study were excluded.
Replication	Replication is not relevant to this study.
Randomization	Randomization is not relevant to this study.
Blinding	Blinding is not relevant to this study.

Reporting for specific materials, systems and methods

We require information from authors about some types of materials, experimental systems and methods used in many studies. Here, indicate whether each material, system or method listed is relevant to your study. If you are not sure if a list item applies to your research, read the appropriate section before selecting a response.

Materials & experimental systems

n/a	Involved in the study	
☒	☐	Antibodies
☒	☐	Eukaryotic cell lines
☒	☐	Palaeontology and archaeology
☒	☐	Animals and other organisms
☐	☒	Human research participants
☒	☐	Clinical data
☒	☐	Dual use research of concern

Methods

n/a	Involved in the study	
☒	☐	ChIP-seq
☒	☐	Flow cytometry
☒	☐	MRI-based neuroimaging

Human research participants

Policy information about studies involving human research participants

Population characteristics	This is a previously reported cohort of 104 predominantly Chinese individuals with suspected paediatric-onset genetic disorder who has performed exome sequencing in 2013-2017 (PMID: 30109123).
Recruitment	All 104 individuals in this cohort were recontacted for this follow-up study. Appropriate consent for enrolling into the study has been obtained.
Ethics oversight	Ethics approval was granted by the Institutional Review Board, the University of Hong Kong/Hospital Authority Hong Kong West Cluster (UW 12-211).

Note that full information on the approval of the study protocol must also be provided in the manuscript.
